# Design and Optimization of a Custom-Made Six-Bar Exoskeleton for Pulp Pinch Grasp Rehabilitation in Stroke Patients

**DOI:** 10.3390/biomimetics9100616

**Published:** 2024-10-11

**Authors:** Javier Andrés-Esperanza, José L. Iserte-Vilar, Víctor Roda-Casanova

**Affiliations:** Department of Mechanical Engineering and Construction, Universitat Jaume I, 12071 Castellón, Spain; jiserte@uji.es (J.L.I.-V.); vroda@uji.es (V.R.-C.)

**Keywords:** exoskeleton, optimization, rehabilitation

## Abstract

Stroke often causes neuromotor disabilities, impacting index finger function in daily activities. Due to the role of repetitive, even passive, finger movements in neuromuscular re-education and spasticity control, this study aims to design a rehabilitation exoskeleton based on the pulp pinch movement. The exoskeleton uses an underactuated RML topology with a single degree of mobility, customized from 3D scans of the patient’s hand. It consists of eight links, incorporating two consecutive four-bar mechanisms and the third inversion of a crank–slider. A two-stage genetic optimization was applied, first to the location of the intermediate joint between the two four-bar mechanisms and later to the remaining dimensions. A targeted genetic optimization process monitored two quality metrics: average mechanical advantage from extension to flexion, and its variability. By analyzing the relationship between these metrics and key parameters at different synthesis stages, the population evaluated is reduced by up to 96.2%, compared to previous studies for the same problem. This custom-fit exoskeleton uses a small linear actuator to deliver a stable 12.45 N force to the fingertip with near-constant mechanical advantage during flexion. It enables repetitive pulp pinch movements in a flaccid finger, improving rehabilitation consistency and facilitating home-based therapy.

## 1. Introduction

In the last 20 years, the number of stroke cases among people aged 20 to 64 years has increased by 25%. Stroke often leads to neuromotor disabilities that can impair finger movement. Since finger movement is crucial for basic activities of daily life (ADL), there is significant motivation to prioritize finger rehabilitation following injury or stroke [1]. It is well established that repetitive flexion and extension movements of the fingers, even when performed passively, can promote neuromuscular re-education, help prevent spasticity, and manage pain associated with hand paralysis resulting from acquired brain damage [2,3].

In view of the consequences and to help with post-stroke recovery, autonomous solutions focused on the functional rehabilitation of the hand’s prehensile capabilities are being developed, among which are hand exoskeletons. Exoskeletons may offer a more effective option than end-effector robot-assisted devices for treating finger motor impairment in stroke patients [4]. These exoskeletons are structures that totally or partially cover the affected hand, and according to the functions they can perform, a distinction can be made between augmentative or assistive (improving capabilities for the execution of specific tasks) for teleoperation (haptic devices oriented to virtual models) and hand exoskeletons for rehabilitation (RHEx) [5]. The RHEx try to recover the motor abilities of patients by forcing repetitive actions on the affected joints: the most common ones are opening and closing the fingers. In this way, it is possible to lengthen the sessions and reduce the physicians’ permanent need for assistance. RHEx are not necessarily portable or lightweight; they can be fixed in a certain position. Even so, they are more versatile if they are portable because the patient can take them home to continue rehabilitation on their own. Moreover, the rapid progress of flexible electronics aligns with the growing demand for portable smart exoskeletons embedded with skin-interfacing flexible electronics (e-skin) [2,6,7] provide fine-grained control and feedback RHEx, allowing for continuous and real-time monitoring of physiological signals. This helps users regain movement and improve strength in affected extremities. Still, the lightness and pragmatism of the exoskeleton topology must be optimized.

The goal population largely conditions the suitability of an RHEx design. The movements allowed by the RHEx should follow patients’ behavior naturally and without generating discomfort. The literature [5] compiles characteristics and criteria that should guide the design of RHEx. However, the evaluations presented in RHEx proposals only include partial aspects, on a few subjects, and rarely patients [2]. In this sense, the collaboration with rehabilitators in the context of the present research (DERAPPI project [8]) has made clear the characteristics of the patient that are suitable for therapy with RHEx:

- Plegic hand (MRC 0 [9,10] in all muscle groups of the hand).
- Flaccid hand (spasticity up to MAS 1 [11] would be admitted).
- Absence of rigidity in the fingers. In case of some rigidity, it cannot interfere with normal intelligent grasping movement.
- Skin is intact and there is no edema at the level of the hand.
- Cognitive status enough to follow simple instructions.
- Behaviorally stable.

For certain patients, it is essential to restrict the range of motion of specific joints to prevent articular degeneration. This goal is difficult to achieve with some RHEx designs inspired by soft robotics. Additionally, there is a lack of devices on the market that simultaneously target the mobilization of the index finger and are easy for either the physician or the patient to place on the hand [12]. Focusing on the recovery of the index finger is crucial since it plays a key role in many grasps and ADL. Regarding RHEx control, devices operated by a pushbutton activated by the healthy hand or programmed to function in a loop are highly suitable for stroke rehabilitation. Applying the concepts of robustness and simplicity makes it desirable to be able to use a simple control rather than having to command and coordinate several actions. The idea behind underactuation in robotics is to use an ingenious mechanical system that can adapt to the requirements of a grasp or a trajectory with one actuator [13]. This mechanical intelligence is commonly found in mechanical linkages where the different link lengths and joint types are determined at the design stage to follow a particular trajectory or adopt a particular posture. If this trajectory is entirely predetermined, then only one DOF suffices to follow it. Moreover, using a single actuator contributes to maintaining the lightweight and compact design of the device, which is desirable in a portable RHEx; therefore, the concept of using a single actuator for underactuation also merits consideration.

**Figure 1:**
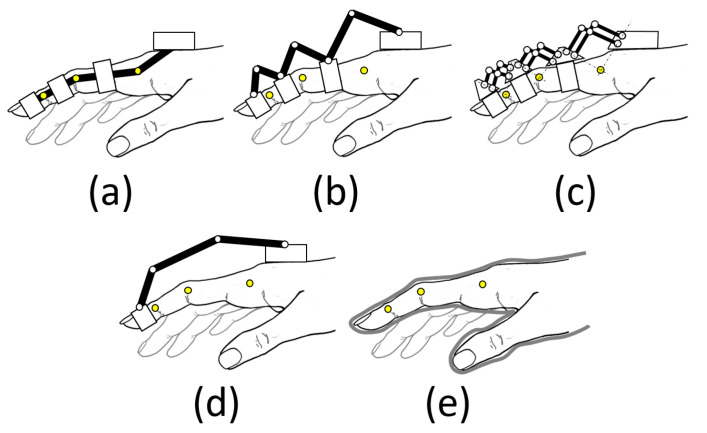
Schematics of various RHEx topologies for transmitting motion to the patient’s fingers: (**a**) matched axes; (**b**) redundant linkage; (**c**) remote center of motion; (**d**) base-to-distal; and (**e**) compliant [12,14].

Several RHEx topologies can be discerned [12,14]:

(i) Those restraining each phalanx (Figure 1a–c), using mechanisms to match the axes of rotation with those of the phalanges. They strictly limit spasticity during rehabilitation. However, the complexity of the design increases as higher finger mobility is sought.
(ii) Those that hold only the fingertip (base-to-distal topology, Figure 1d), with the advantage that their design is simpler, as they do not have the restriction of matching the axes of rotation, so they can be lighter as well. Note that the three degrees of freedom that position and orient the distal phalange (DP) within the plane of flexion of the finger univocally condition the three degrees of freedom provided by the three joints: metacarpophalangeal (MCP), proximal, and distal interphalangeal (PIP, and DIP). However, the movement of the finger is less controlled, regarding the variety of exercises that can be performed by acting on the individual joints.
(iii) Those that fit over the entire finger, glove type (Figure 1e), using flexible and lightweight materials that adapt to the hand (soft robotics). They are less cumbersome and less expensive, but care must be taken in the forces applied.

The choice between these approaches depends on the specific goals of the device and the patient’s needs in terms of rehabilitation. In the case of soft robotics (iii), having no rigid joints to guide torques and forces, the user’s skeleton becomes the guiding structure, and it may not be an appropriate choice in dealing with the patient’s spasticity. The RHEx based on articulated mechanisms guide the movement based on a guiding structure (an articulated linkage that performs the function of guiding the movement) and a guiding chain (a system that transmits the actuation, usually a Bowden cable, electrical, pneumatic, or a system of additional links to the first ones [15]).

Control for RHEx can be relatively simple if they are aimed at task-specific training, allowing for passive control that overlooks the patient. For case (i), if independent control is desired for each degree of mobility of the hand, individual actuators will be required to adjust and control the movements of each finger independently. However, the design of the exoskeleton can be greatly simplified, and the cost of the device can be reduced by using sub-actuated designs, especially in case (ii), which makes them very economical and easy to transport. In this case, the RHEx may actuate on some degrees of freedom, leaving the others to move passively, i.e., allowing certain joints to freely adopt the position that best suits them, within their anatomical ranges of motion, to achieve the posture demanded by the exoskeleton.

In cases (i) and (ii), the location of the mechanism can be either dorsal (located on the dorsal part of the fingers, Figure 1b–d), palmar (on the palmar part of the fingers, hardly used because they interfere with interacting with objects [16]), or lateral (Figure 1a). Lateral devices leave the palm of the hand free and therefore allow objects to be grasped. In addition, they allow patients to see their hands, which is important for visualizing progress during rehabilitation. In any of the three arrangements, the mechanism is attached to the hand via Velcro^®^, flexible straps, or thimbles.

In recent years, different RHEx designs have been proposed in the literature [2,14,15,17], and there are already some commercial designs for diverse purposes [16,18]. There are very few RHEx reported in the literature that reach TRL level 9 [19,20], and, even though much research is being carried out on them, they are still far from being a practical solution. In a recent extensive literature search on the design and optimization of exoskeletons (over 722 studies from 2017 to 2023), it was shown that evolutionary computation (EC) methods are the most frequently used (genetic algorithms, particle swarm optimization, differential evolution, and evolutionary strategies) compared to other non-EC [21]. This outcome is anticipated given EC methods’ numerous attributes, such as the ability to update multiple solutions simultaneously (population-based), their independence from gradient information, rapid convergence, and their capacity to tackle mixed optimization problems, including multimodal and multi-objective scenarios, to optimize different metrics (mainly force transmission (FT), workspace, weight, and size). However, this research found only two studies in which EC techniques were used for hand exoskeletons: one with an assistive application [22] and another for rehabilitation (RHEx) [23]. Close to these dates, we also found other four studies using EC for improving an RHEx [24,25,26,27]. Table 1 summarizes the studies related to the optimization of hand exoskeletons from 2014 to 2024.

Du et al. [23], and Sarac et al. [27], used the Single-Objective Genetic Algorithm [30] to optimize the FT, whereas Iqbal et al. [24] used a Weight-Based Genetic Algorithm (WBGA) [21], the simplest classical method of solving multi-objective optimization problems, which considered factors like kinematic mapping, collision avoidance, and a global isotropy index, and FT. Vanteddu et al. [25] also used WBGA, the cost function being the weighted sum of errors for the end position of the grasp trajectory between the kinematic model and the HUST (Huazhong University of Science and Technology) dataset concerning the joint angles of a natural human finger. Li et al. [26] proposed a multi-parameter multi-objective optimization method (namely, the Elitist Non-Dominated Sorting Genetic Algorithm (NSGA–II) [28,29]) to enhance the three global manipulability measures simultaneously in a wearable index finger rehabilitation exoskeleton.

In those works, which used FT as the metric to observe in EC methods, the improvements achieved in either the method or the results are poorly described: Li et al. [22] worked with two functions, one to obtain the three contact forces via three phalanges to the object, distributed as evenly as possible, and another to maximize the sum of the forces exerted on the index finger phalanges by the proposed assistive exoskeleton. It is important to note that it only focuses on the objective in the final grasping, not on the entire finger travel as required in rehabilitation therapy. Their EC worked with a population of only 100 individuals, with a stall generation limit set to 500. Du et al. [23] focused on the transmission angle, a geometrical concept related to FT and mechanism geometry, but they do not detail the improvements achieved after 1000 generations (without detailing the population). Since the objective is to improve FT, it would be much more logical to work on concepts such as mechanical advantage. Iqbal et al. [24] do not describe their Overall Impact Factor used as a weighted optimization function, and how the FT is included. Sarac et al. [27] used an optimization function based on the sum of the squared torques applied to the MCP and PIP joints in their design. However, it is unclear how this function accounted for the full range of motion and the characteristics of the population studied. No studies were found using multi-parameter multi-objective optimization methods targeting metrics directly related to optimal FT for RHEx, in which the mechanical advantage is highly variable, and it is important to maintain a proper value for the entire range of motion, not only the final posture.

The goal of this work is to design a six-bar linkage intended for implementation in a hand-held RHEx, which can drive the DP from its initial to its final position, mirroring a pulp pinch grasp movement (aka. tip pinch [42]). This grasp has been documented as the most frequent in ADL [43,44]. The study focuses on six-bar mechanisms because this topology is of interest for the conception of RHEx to be placed either on the lateral or on the dorsal side of the index finger, partially mimicking the movement of the phalanges. Extending the design to other fingers would cause a single dorsal four-bar mechanism to interfere with the finger’s dorsum. In the authors’ previous works [45,46], a structured exploration of the design space for this linkage was performed to obtain good initial values for subsequent genetic optimization, following a methodology similar to that proposed in [47]. In those works, the design space for this linkage considered 10 free parameters. Three different values were selected for each of these variables, resulting in 59,049 different versions of the six-bar linkage. All these combinations were explored, and only 11,533 were regarded as valid. The maximum mechanical advantage was calculated for each valid solution, which was then used to sort and select the optimal design based on it.

In the present work, and for the optimization, we propose a targeted genetic scheme that reduces the number of generational calculations until an optimal solution is found. This scheme consists of two stages with two different targets, namely: first, the optimal location of a specific joint, and then the optimal dimensions of the linkage. Each one of the stages observed two quality metrics: averaged mechanical advantage along the travel, and its variability. These metrics are often conflicting, meaning improving one may worsen another. The solutions to multi-objective problems are frequently expressed as a set of Pareto optimal solutions, where no solution can be said to be better than another without considering trade-offs. The methodology here proposed lies in the field of ranking-based selection strategies with hierarchical objective prioritization. While it does not directly correspond to a widely recognized standard technique, it can be considered a variation of methods like NSGA-II or SPEA2 (Strength Pareto Evolutionary Algorithm 2) [48], where objectives are given relative importance instead of being treated entirely equally. According to our knowledge, it has never been applied to optimize the design of RHEx with such metrics, both the mechanical advantage and its variability, both related to FT.

The following section lists the design requirements from the scope of rehabilitation. Section 2, after meeting the specific requirements of the patient (dimensions and range of movement of the DP) to customize the proposed solution, delves into the mechanism topology selected for the design of the RHEx, as well as the methodology followed for its dimensional synthesis and optimization. Section 3 shows the results obtained in the current research. The final model is proposed at the end of Section 3. The last two sections are devoted to discussion and conclusions.

### 1.1. Design Requirements

In the context of the DERAPPI project [8], rehabilitators from the Brain Injury Unit of the Hospital La Magdalena (Castellón, Spain) made a detailed list of clinical needs for an RHEx to be integrated into the routine of clinical practice, for the rehabilitation of pulp pinch in subacute stroke patients:

- The thumb should be able to perform the opposition movement, or, if in its defect, keep it in opposition to the index finger, to be able to perform the bidigital pulp pinch.
- The RHEx should leave the palmar side of the hand and fingers clear so as not to interfere with the sensory stimuli generated when grasping an object and not to excessively favor the flexor pattern of the plegic hand.
- The RHEx should leave the wrist joint free to allow for the tenodesis effect in case it is present in the patient, namely, when finger movement involves passive wrist movement.
- Leave the pulp of the thumb and index finger free to allow tactile feedback during movement.
- The RHEx design must be accessible to different hand sizes.

For the RHEx to be safe in stroke patients, some biomechanical restrictions must also be verified in relation to the forces applied by the device: the use of linear actuators is justified compared to a more economical hydraulic system, since they allow for more detailed control of the movement, joint ranges, and pressure exerted in the pulp pinch movement.

From the observations made, it was concluded that the minimum force required to guide the distal phalanx of the index finger should be approximately (not less than) 5 N (≈0.5 kgf) in a closing sequence of 1 s duration.

As listed above, thumb abduction is required to perform the pulp pinch. In the abducted position, the thumb can oppose the fingertips [42]. Since including this movement would add complexity and therefore weight and cost to the exoskeleton, it was decided that the exoskeleton structure itself would keep the thumb permanently in opposition to allow the pinch without any specific actuators for it, thus reducing the number of actuators needed without compromising the rehabilitation movement.

## 2. Materials and Methods

### 2.1. Characterization of the Two-Finger Gripper Motion

The present RHEx is intended for patients with reduced hand mobility. In relation to the functional range of motion, there are differences among patients, reaching up to 40 degrees of difference in some cases at the MCP, PIP, and DIP joints’ flexion angles [49] due to the different anthropometry between subjects. It can be settled that the most appropriate approach is a customization of the dimensions of the mechanism to allow for a movement as close as possible to the natural movement of each patient. In this context, the use of contact measurement instruments (instrumented gloves, dimensional measurements) is not feasible for this type of patient [50]. Instead, 3D scanning has been shown to be the least invasive with the nature of the patient, as well as providing adequate detail of the anatomy and dimensions of the hand for subsequent adaptation of the mechanism, compared to other abovementioned methods.

**Figure 2:**
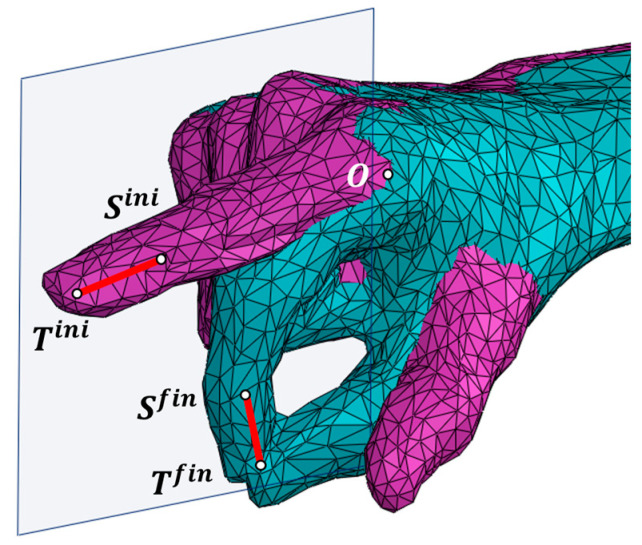
Overlapped 3D-scanned hand in two different postures: extended index (pink) and pulp pinch grasp (green).

Figure 2 shows the 3D-scanned hand of a stroke patient in the two boundary postures of a pulp pinch grasp movement, with the starting posture being the functional resting position [51]. Thumb motion during the pulp pinch has been measured by videogrammetry in numerous studies, indicating that healthy subjects maintain the thumb in opposition throughout the pinch [51,52]. Jahn et al. [53] identified three distinct phases that the joints progress through during the execution of the pulp pinch: the initiation phase, the preshaping phase, and the pinch phase. In the initiation phase, little to no movement occurs. It is important to note that most rehabilitation exercises are designed to begin from the functional resting position [51], as is assumed in this work (Figure 2): the wrist is positioned between 10° and 30° of extension, the thumb is in opposition and abduction, and the finger joints are in semiflexion [54]. This posture is often referenced by splints designed to support the hand in such impairment conditions [55].

The preshaping phase accounts for the majority of the movement and begins with the first noticeable increase in motion. The pinch phase starts when the slope decreases as the movement comes to a stop. Thus, the preshaping phase is the focus of gesture rehabilitation as it predisposes and defines the intended grasp. According to the physicians involved in this study, with the exoskeleton’s goal being rehabilitation rather than assistance with ADLs, it was observed that the thumb’s range of motion from the functional resting position is minimal. In fact, the index is the first finger to have all joints actively engaged during the preshaping phase, followed by the thumb. The preshaping phase ends in a proximal-to-distal pattern for the index finger, while in the thumb, the last joint to become actively involved is the MCP, which is primarily predisposed when in the functional resting position [53]. Therefore, in this context, it is considered appropriate for rehabilitation to focus initially on the movement of the index finger, with the thumb showing minimal motion or being stabilized within a splint.

The initial position of the DP of the index finger is defined by points $S^{ini}$ (DIP joint) and $T^{ini}$ (tip of the finger), and the final position is defined by points $S^{fin}$ and $T^{fin}$. Point O is set at the MCP joint on the index as the connection of the first mobile link of the RHEx with the palm (ground bar). Finally, a working plane is set as parallel to the one fitted to {$S^{ini},T^{ini},S^{fin},T^{fin}$} data, minimizing the sum of the squared distances between the points and the plane through a least squares approach [56], at a distance so that it does not interfere with the hand. In this plane, the RHEx will perform its motion. To proceed with the design, we obtain orthogonal projections on this plane of the distal segment (ST) at the initial and final positions and of the O point.

### 2.2. Linkage Topology

**Figure 3:**
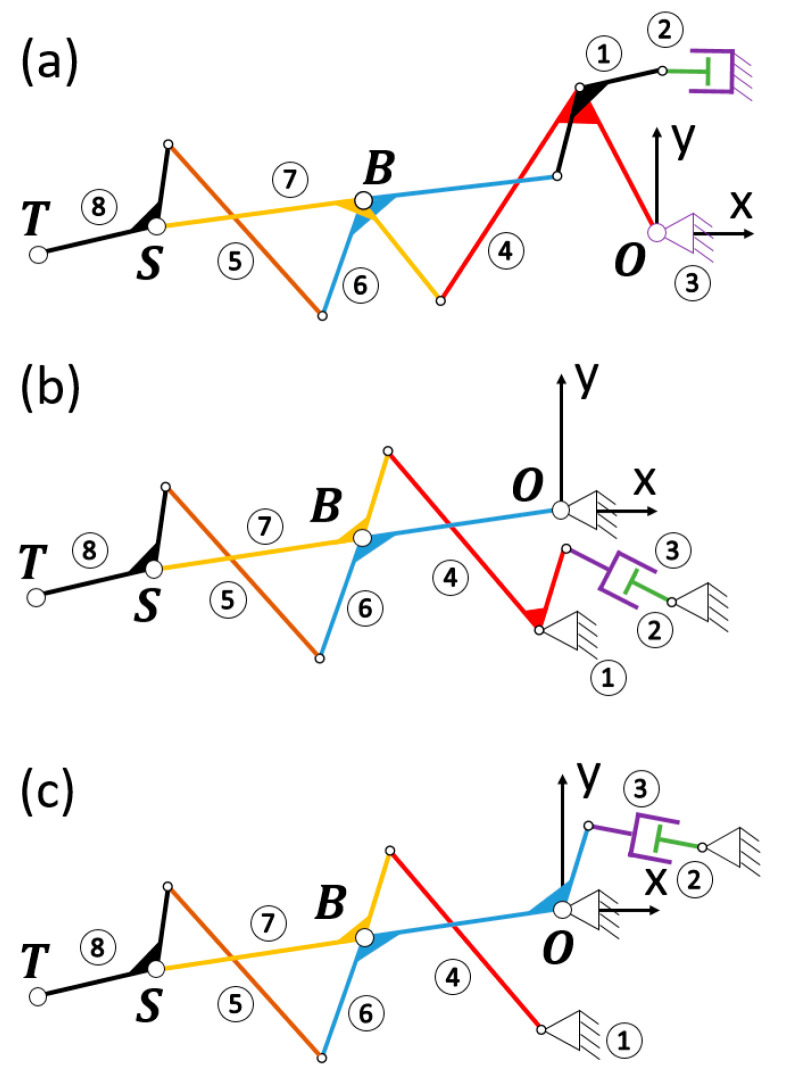
TBM and RML mechanisms compared: (**a**) TBM consisting of two four-bar mechanisms and a crank–slider in the first inversion; (**b**) change of the crank–slider to its third inversion; (**c**) resulting RML mechanism having selected link 4 as the coupler of the crank–slider.

Multiple four-bar linkages, made up of a series of concatenated crossed four-bar mechanisms, are complex assemblies where the motion is transferred through a sequence of interconnected four-bar linkages, enabling the design of intricate mechanical systems with sophisticated movement capabilities. This versatility has led to significant innovation and a variety of designs. Our research group has experience in applying different topologies for the design of prosthetic devices, such as the Toronto/Bloorview/MacMillan (TBM) [57,58], consisting of two coupled four-bar mechanisms. The proximal one is coupled with a crank–slider mechanism in its first inversion (cylinder being fixed), with the crank being part of link 4 and the coupler being part of link 1 (see Figure 3a). This results in having a linear actuator as input, and the proximal four-bar mechanism involves links 1-4-6-7. This inversion of the crank–slider implies having the linear actuator integrated within the ground bar (links 1 and 2 at the dorsum of the hand). The RML (Robotics and Mechatronics Lab of the Virginia Tech University, Blacksburg, Virginia, United States) [59] has a similar topology, although the crank–slider providing a linear input is in its third inversion (the coupler link (1) of the crank–slider is fixed). In this mechanism, the proximal four-bar mechanism also involves links 1-4-6-7, although link 1 is the ground at the dorsum of the hand. The third inversion of the crank–slider gives the designer more freedom to optimize the location of the actuator for improved performance (see Section 2.3.3). In addition, the crank is an extension of link 6 (Figure 3c) instead of link 4 (Figure 3b), which becomes a quaternary link. This last variation has the advantage of leaving more spare space at the level of the hand, preventing any interaction and allowing the user better visual feedback. For all these reasons, RML topology was used for the forthcoming RHEx design. All these topologies have one degree of mobility for the 8 links involved.

As stated earlier, the RML, as the topology here studied, has been used for different proposals of RHEx [37,59,60,61], although no optimization has been conducted under the requirement of an optimal force transmission [25].

### 2.3. RHEx Design

#### 2.3.1. Dimensional Synthesis

**Figure 4:**
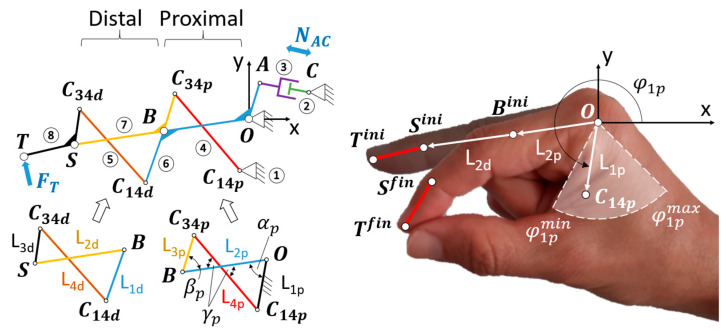
(**Left**) nomenclature of the segments that form the concatenated four-bar mechanisms; (**Right**) region where to locate point $C_{14p}$, delimited by the maximum and minimum values of $\varphi_{1p}$ and $L_{1p}$.

In the process of synthesizing the two four-bar mechanisms within the linkage, let us consider a recurrent nomenclature for both, the proximal and distal ones concerning the patient’s hand (see Figure 4). The segments of each four-bar mechanism are named from $L_{1}$ to $L_{4}$, indicating with the subscript p or d whether they belong to the proximal or distal mechanism, respectively. Segments $L_{2p}$ (belonging to link 6) and $L_{2d}$ (belonging to link 7) are articulated in B, where both four-bar mechanisms are concatenated through the relative movement of segments $L_{1d}$ (also belonging to link 6) and $L_{3p}$ (also belonging to link 7).

The synthesis to be performed involves two positions (initial and final) of the ST segment corresponding to the pulp pinch grasp (Figure 4). As has been mentioned before, let us designate $S^{ini}$ and $T^{ini}$ as the points that make up this segment in the initial position. Analogously, $S^{fin}$ and $T^{fin}$ correspond to the final posture.

**Figure 5:**
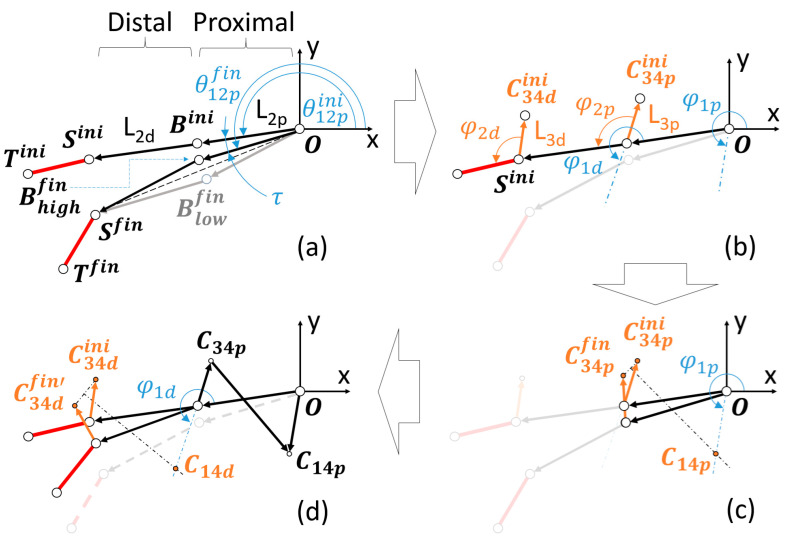
(**a**) Location of $B^{ini}$ and determination of the lengths $L_{2p}$ and $L_{2d}$ and of $B^{fin}$. (**b**) Selection of the design parameters: $L_{3p}$, ${\varphi_{2p},\varphi_{1p},L}_{3d}$, $\varphi_{2d}$, $\varphi_{1d}$. (**c**) Determination of $C_{14p}$ and therefore of $L_{1p}$ and $L_{4p}$. (**d**) Superimposition of $L_{2p}$ bars in both postures to obtain $C_{14d}$, $L_{1d}$, and $L_{4d}$ in an analogous procedure.

By the inherent nature of the flexion of the finger, the distance between $S^{ini}$ and O is longer than the distance between $S^{fin}$ and O. Therefore, any point $B^{ini}$ that divides the line $S^{ini}O$ into the two segments $L_{2p}$ and $L_{2d}$, for the construction of the proximal and distal four-bar mechanisms, guarantees the existence of two possible solutions for the flexed finger posture, namely, $B_{high}^{fin}$ and $B_{low}^{fin}$ (see Figure 5a). Of these two, for mimicry with the two-finger pincer movement, $B_{high}^{fin}$ will be considered to continue the synthesis procedure (for the sake of brevity, $B^{fin}$).

Without loss of generality and for further optimization, we started considering a $B^{ini}$ corresponding to the midpoint of the segment $S^{ini}O$. With the lengths $L_{2p}$ and $L_{2d}$ known, values can be proposed for the following design parameters: $L_{3p}$, ${\varphi_{2p},\varphi_{1p},L}_{3d}$, $\varphi_{2d}$, $\varphi_{1d}$ (see Figure 5b). Due to the functional purpose of the RHEx, maximum and minimum values have been established for these parameters, as shown in Table 2. The first three correspond to one length and two angles of the proximal four-bar mechanism: length $L_{3p}$ and angle $\varphi_{2p}$ (the latter measured with respect to $L_{2d}$) allow for the locating of the $C_{34p}$ joint between bars $L_{3p}$ and $L_{4p}$ (in both positions corresponding to extended finger, $C_{34p}^{ini}$, and flexed, $C_{34p}^{fin}$). The angle $\varphi_{1p}$ with respect to the ground bar stipulates the line on which the joint $C_{14p}$, between bars $L_{1p}$ and $L_{4p}$, will be located. Figure 4 (right) shows the region where point $C_{14p}$ should be located, delimited by the maximum and minimum values of $\varphi_{1p}$ and $L_{1p}$ chosen after observing the anatomical dimensions of the patient’s hand. More specifically, the limit values of $\varphi_{1p}$ are specified in Table 2, while any value obtained after the synthesis for $L_{1p}$, with a maximum value of 50 mm, is considered valid. This value is based on the subjectivity of not exceeding the margins of the first dorsal interosseous of the patient’s hand in the pulp pinch posture.

In a graphical synthesis process, $C_{14p}$ is determined by drawing the bisector of the segment that joins the representation joints $C_{34p}^{ini}$ and $C_{34p}^{fin}$ (see Figure 5c). Consequently, the lengths of $L_{1p}$ and $L_{4p}$ are also determined.

We proceed in an analogous way to obtain the distal four-bar mechanism, being $\varphi_{2d}$ defined with respect to the distal segment (ST). Previously, it was necessary to make a rotation of τ to superimpose the $L_{2p}$ bars of the mechanism in both postures (initial and final) and thus work with $C_{34d}^{fin\prime }$ (see Figure 5a,d). Results of $L_{1d}$ with a maximum value of 50 mm were considered valid. Again, this value is subjectively eligible to avoid invading the palmar space in excess during any grasp.

#### 2.3.2. Optimization Algorithm

The synthesis procedure described in the previous section was programmed in Matlab^®^ R2018b. This same platform was maintained for the subsequent programming of the optimization process, which requires recurrent repetitions of the synthesis process as well as the selection of solutions based on the metrics described below. No Matlab^®^ optimization library was used in order to have full control of the design of operations.

**Figure 6:**
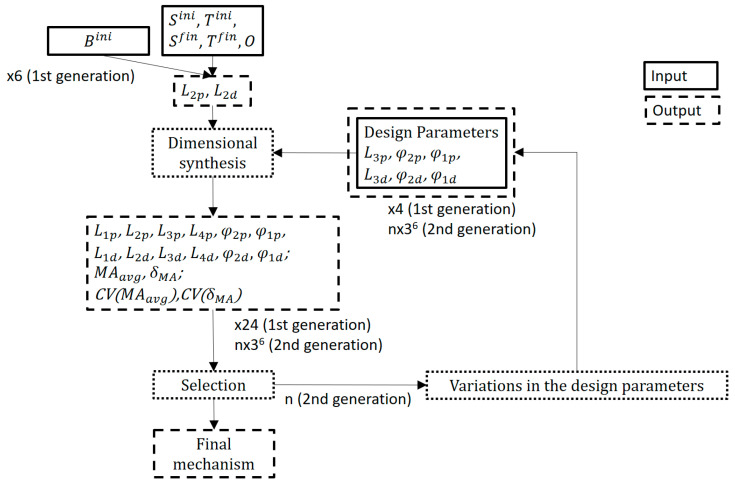
Two generations were studied, for a total of 2211 mechanisms.

A genetic scheme was adopted for the present study, in two stages (thus having two generations) with two different targets. The first step was to provide discretion to the optimization in order to reduce the study population. To this end, the relevance of the location of the B-joint ($B^{ini},$ Figure 5a) between the proximal and distal four-bar mechanisms was studied, resulting in a pre-selection. Next, the influence of the variation in the values (dimensions) of the different lengths and angles set for the synthesis of the mechanism was investigated. Figure 6 summarizes the two generations in which, in the first instance, the $B^{ini}$ localization was optimized by studying a population of 24 mechanisms, on which a first selection was made. In the second generation, the mechanisms resulting from varying certain design parameters in the selected mechanisms were studied.

As objective functions, the mean value of the mechanical advantage for the entire range of motion of the exoskeleton, as well as its level of variation with respect to this mean value, were observed. Heuristic multi-objective approaches with dynamic objective prioritization refer to optimization techniques where multiple objective functions are optimized, not treating all objective functions equally (as in typical Pareto-based methods). Instead, the algorithm prioritizes certain objectives over others (hierarchical preference or dynamic prioritization), often in stages, or dynamically (where the objective importance changes based on some conditions or measures). These hierarchical approaches have the advantages of (i) flexibility, as they allow different treatment of objectives based on the stage of optimization or problem-specific priorities; (ii) adaptability, as they help balance exploration and exploitation in complex sceneries; and (iii) simplicity, as they provide an intuitive way based on heuristics [62,63]. Both generations and their optimization details reflected in Figure 6 are explained in detail hereunder.

1. First Generation: Optimization of the location of the joint B

**Figure 7:**
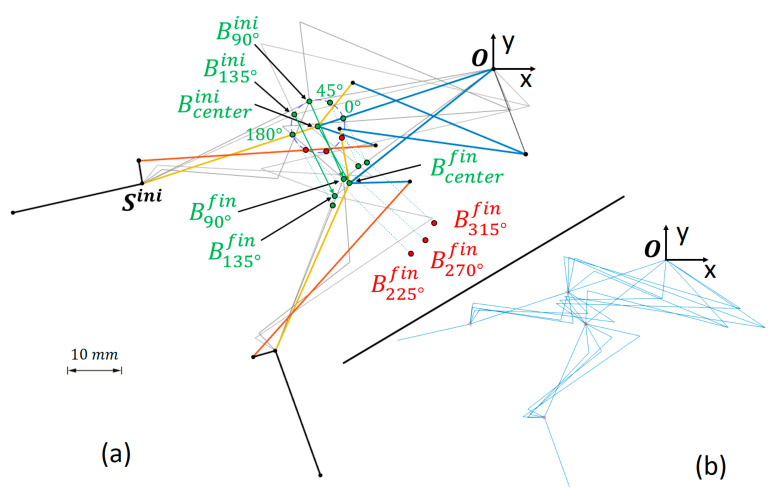
(**a**) The figure illustrates how some initial re-locations of $B^{ini}$ around the initial hypothesis ($B_{center}^{ini}$) lead to configurations where $B^{fin}$ ends up above (accepted solutions, in green) or below (rejected solutions, in red) the line $S^{ini}O$. In grey in the background, two additional mechanisms generated under the $B_{90{}^{\circ}}^{ini}$ and $B_{135{}^{\circ}}^{ini}$ hypotheses are exemplified. (**b**) Four proposals generated after dimensional synthesis for the same $B_{center}^{ini}$, with random values for the parameters of Table 2.

In the first stage, the selection of point $B^{ini}$ was reconsidered. For this, and in the posture corresponding to the extended finger (Figure 4-right), we could determine a series of possible relocations of point $B^{ini}$ in polar coordinates, namely, describing a circumference around the first $B^{ini}$-hypothesis, at a radius R=5 mm, and every 45°. In any of these other relocations of $B^{ini}$, the initial posture is no longer undetermined, and it is easy to observe that those options for point $B^{ini}$ that result in a configuration above the line $S^{ini}O$ maintain that configuration throughout the movement of the mechanism. The same is true for relocations of $B^{ini}$ that result in a configuration below the line $S^{ini}O$. These solutions are discarded because they offer final configurations (flexed finger) that could interfere with some of the rehabilitation activities (see $B_{225{}^{\circ}}^{fin}$, $B_{270{}^{\circ}}^{fin}$, and $B_{315{}^{\circ}}^{fin}$ in Figure 7a).

The initial synthesis process, explained in Section 2.3.1, is valid for any of these relocations of $B^{ini}$. Given the complexity of the mechanism, it was likely that any attempt to select values between the limits of Table 2 would result in a non-existing solution in either of the four-bar mechanisms. Instead, it was decided to generate random values between these limits, guaranteeing the existence of solutions through the stages of the synthesis (proximal four-bar, followed by the distal one) by performing iterative calculations until a valid solution was found. Four simulations were run for each hypothesized $B^{ini}$ point to have a sufficiently diverse population (see Figure 7b).

To assess the performance of each design, two quality metrics were evaluated: the averaged mechanical advantage (${MA}_{avg}$) over the entire RHEx travel, and the degree of irregularity of the mechanical advantage ($\delta_{MA}$) over the same travel, defined as

(1) $${MA}_{avg}=\frac{1}{\theta_{12p}^{fin}-\theta_{12p}^{ini}}\cdot \int_{\theta_{12}^{ini}}^{\theta_{12}^{fin}}MA\left(\theta_{12p}\right)\cdot d\theta_{12p},$$

(2) $$\delta_{MA}(\%)=\frac{{MA}_{max}-{MA}_{min}}{{MA}_{avg}}\cdot 100.$$

For each mechanism, and for the calculation of the ${MA}_{avg}$, the mechanical advantage (MA) was evaluated at four equidistant positions along the $\theta_{12p}$ travel (see Figure 5a), including the initial and final postures. This allowed for the interpolating of a third-degree polynomial function $MA\left(\theta_{12p}\right)$ for each mechanism. The evaluation of the MA at each of the four postures was performed using approximate methods, namely (see Figure 4-left): (i) without loss of generality, point A was considered at a distance of 10 mm from O; (ii) concerning the posture being studied at a $\theta_{12p}$ value, displacements of point A ($∆s_{A}$) and point T ($∆s_{T}$) were evaluated (these displacements were measured from the posture resulting from a decrease ${∆\theta }_{12p}$ to the posture resulting from an increase ${∆\theta }_{12p}$ (${∆\theta }_{12p}=10^{-5}$ rad)); and (iii) MA for the referred posture was calculated as

(3) $$F_{T}\cdot \dot{s_{T}}=N_{AC}\cdot \dot{s_{A}}\to MA=\frac{F_{T}}{N_{AC}}\approx \frac{∆s_{A}}{∆s_{T}}.$$

For the calculation of Equation (3), we assume an ideal mechanism so the power developed at point A by the linear actuator ($N_{AC}$) must coincide with the power developed at point T.

When evaluating the mechanical advantage, it should be noted that all the dimensions of the mechanism were already known, after the synthesis of the previous section. Therefore, the displacement $∆s_{T}$ was estimated from the variation undergone by the concatenated four-bar mechanisms, caused by the rotation of A around O, here approximated to $∆s_{A}$. Without loss of generality, for the proximal mechanism depicted in Figure 4-left, it follows that

(4) $$\vec{OB}+\vec{OC_{34p}}+\vec{C_{34p}C_{14p}}-\vec{OC_{14p}}=\vec{0}.$$

Projecting the vectors of the loop into a local coordinate system with the abscissa axis along $L_{2p}$ we have

(5) $$L_{4p}\cdot cos\gamma_{p}=L_{2p}-L_{3p}\cdot cos\beta_{p}-L_{1p}\cdot cos\alpha_{p},$$

(6) $$L_{4p}\cdot sin\gamma_{p}=L_{3p}\cdot sin\beta_{p}+L_{1p}\cdot sin\alpha_{p}.$$

By squaring the Equations (5) and (6), adding them, and using the fundamental trigonometric identities, the dependence on the angle $\gamma_{p}$ is removed. This allows obtaining the value of $\beta_{p}$ as a function of $\alpha_{p}$. The distal four-bar mechanism can be solved analogously. Thus, for each value of $\theta_{12p}$ and its variation (${∆\theta }_{12p}$), the values of $\alpha_{p}$, $\beta_{p}$ and their variations (${∆\alpha }_{p}={-∆\theta }_{12p}$, ${∆\beta }_{p}$) are known univocally. It allowed us to find the displacement required in Equation (3).

With all this, in the first generation, a total of 24 simulations were performed, which comprised the random synthesis of four RHEx proposals for each point $B^{ini}$.

From the number of resulting proposals, the coefficient of variation (CV) of the above quality metrics, Equations (1) and (2), were studied. The CV shows the extent of variability concerning the mean of the population. It is defined as the ratio of the standard deviation (σ) to the mean ($\bar{x}$), namely:

(7) $$CV(\%)=\frac{\sigma }{\bar{x}}\cdot 100,$$

where x denotes the values of the metrics whose CV is evaluated. From the metric with the higher CV, i.e., the higher relative variability in that metric, the first quartile of the population with better values (lower $\delta_{MA}$ or higher ${MA}_{avg}$) was pre-selected. Half of the pre-selected cases with the best values for the other evaluated metric was selected for further optimization.

2. Second Generation: Optimization of the Design Parameters

To assess the influence that the parameters in Table 2 have on the design, the influence of an incremental, null, or decremental variation of each of them was considered (±2 mm for the lengths, ±5° for the angles, in case there was variation). Considering the 6 parameters, it gave a total of $3^{6}=729$ possible combinations for each of the mechanisms selected in the previous section. The dimensional synthesis of each of them was performed as described in Section 2.3.1.

From the total amount of resulting proposals, we observed again the metric with higher CV to pre-select the first quartile of the population with better values (lower $\delta_{MA}$ or higher ${MA}_{avg}$). Finally, the one with better value in the remaining quality metric has been chosen for further development of the RHEx.

In summary, this algorithm can be seen as a hierarchical multi-objective selection approach, where objectives ($\delta_{MA}$ or ${MA}_{avg}$) are prioritized based on their relative variability (CV), followed by selection based on the other objective. This step involves prioritizing one metric over the other based on its variability, introducing a hierarchy in the selection process. The algorithm reinforces the idea that both metrics are important, but with a differential emphasis depending on the variability. Not being a standard method, it can be classified as a heuristic multi-objective approach with dynamic objective prioritization.

#### 2.3.3. Linear Actuator Positioning

Point A depicted in Figure 4-left, where the linear actuator is anchored observes the following design conditions: (i) it performs a rotation around point O; (ii) the rotation covers a circumferential arc whose central angle is the same as the angle τ traveled by the bar $L_{2p}$ in the movement of the RHEx from extension to flexion (Figure 5a); and (iii) the radius of the circumference (OA) is such that the length of the chord ($L_{chord}$) joining the two endpoints of the arc matches with the stroke length of the linear actuator. At the prolongation of this chord, the attachment of the other end of the linear actuator (point C in Figure 4-left) must be found. Also, the chord and the radii joined to the two endpoints of the described arc constitute an isosceles triangle from the resolution of which the value of the radius is determined univocally, namely:

(8) $$OA=\frac{L_{chord}}{2\cdot sin\left(\tau /2\right)}.$$

By observing these three conditions, it is guaranteed that the transmission angle [64] remains as close to the optimum as possible, i.e., moderately close to perpendicular to the radius OA, during the entire stroke. The solutions can still be infinite, and it is necessary to decide the solution to suit the design of the dorsal support of the hand on which the other end of the actuator will articulate.

## 3. Results

### 3.1. Preliminary Designs from the Optimization of the Location of the Joint B

**Figure 8:**
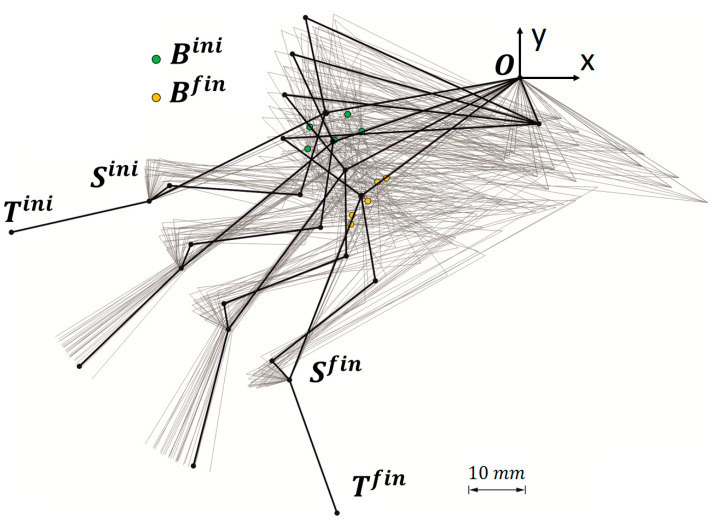
In black, the sequence of motion of the RHEx from extension ($S^{ini}T^{ini})$ to flexion ($S^{fin}T^{fin}$) is shown. The grey background represents the wide spectrum of possible linkages studied, achieved by generating and simulating four mechanisms with varied dimensions for each of the six possible $B^{ini}$ locations detailed in Figure 7a.

Figure 8 shows the wide spectrum of dimensions, within the ranges of the problem (Table 2), that has been addressed by generating and simulating 24 proposals of mechanisms for the different possible $B^{ini}$ locations. They are the result of simulating four mechanisms of varied dimensions based on each of the six possible $B^{ini}$ locations announced in Figure 7a.

**Figure 9:**
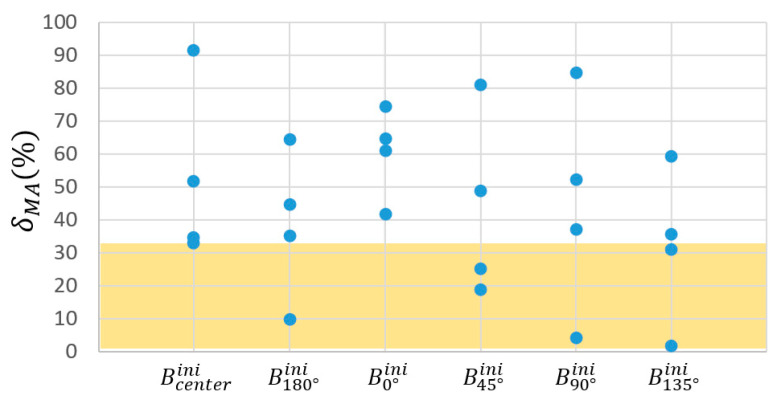
Preselection for the $B^{ini}$ locations, as explained in Section 2.3.2-(1), was conducted based on $\delta_{MA}$ as it exhibits a higher coefficient of variation (CV) compared to ${MA}_{avg}$ across the 24 proposed cases. The colored region highlights the quartile of the population with the lowest $\delta_{MA}$, narrowing down this preselection to six possible RHEx proposals.

**Figure 10:**
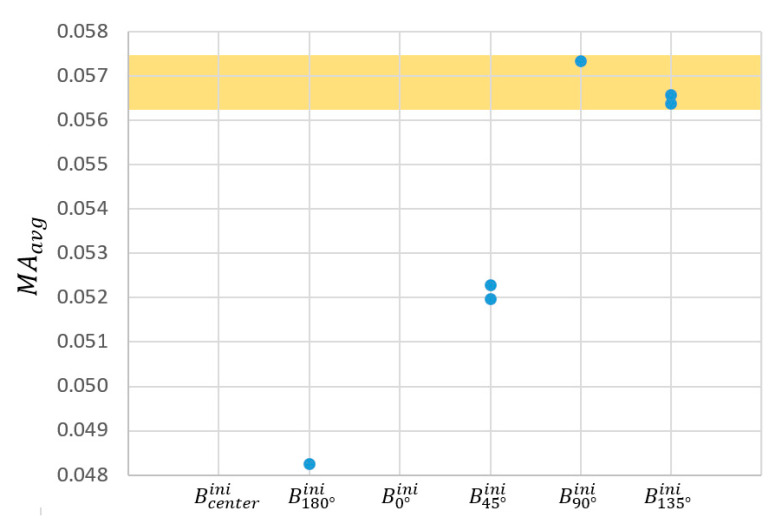
Selection for the optimal $B^{ini}$ locations, as explained in Section 2.3.2-(1), was conducted based on ${MA}_{avg}$ over the previous pre-selection of 6 cases. The colored region highlights the half of the pre-selected cases with the best ${MA}_{avg}$, that is, the three cases selected for further optimization.

The CV of the $\delta_{MA}$ had a value of 52.8%, higher than the CV of the ${MA}_{avg}$ (12.6%). Therefore, according to the procedure explained in Section 2.3.2-(1), the quartile with the lowest $\delta_{MA}$ amongst the 24 cases was considered first. It corresponds to cases represented in Figure 9, namely, one $B_{90{}^{\circ}}^{ini}$ subcase, one $B_{180{}^{\circ}}^{ini}$, and two subcases of $B_{45{}^{\circ}}^{ini}$ and $B_{135{}^{\circ}}^{ini}$ (see Figure 7a). Figure 10 depicts, from the abovementioned cases, the three with the highest ${MA}_{avg}$. In the figures, these variations are grouped after their $B^{ini}$ for the sake of brevity, the subcases from the same $B^{ini}$ being tagged with a and b sub-indexes.

Table 3 shows in detail the results of the dimensional synthesis for the six pre-selected cases and the three finally selected for further study, named $B_{135{}^{\circ}a}^{ini}$, $B_{135{}^{\circ}b}^{ini}$, and $B_{90{}^{\circ}}^{ini}$.

### 3.2. Designs from the Subsequent Optimization of the Design Parameters

**Figure 11:**
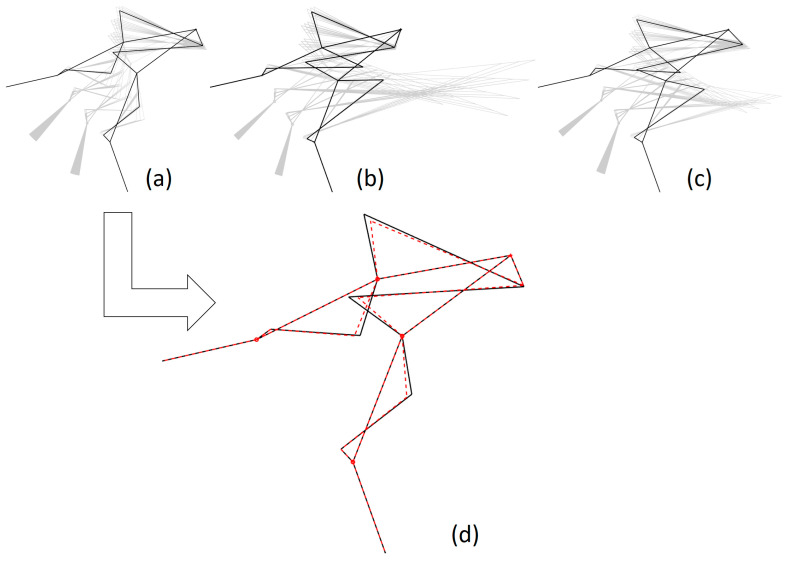
(**a**–**c**) In black, the representation of the three mechanisms selected from Table 3 (after optimization for the location of joint $B^{ini}$, following Section 2.3.2-(1)). The corresponding sets of mechanisms resulting from parameter variations are shown in grey in the background. (**d**) In black, the selected best mechanism after the optimization of the design parameters (following Section 2.3.2-(2)), overlapping with the preliminary design (in red).

Figure 11 summarizes the three best mechanisms over the different locations of $B^{ini}$ and the 3·729=2187 mechanisms resulting from varying the parameters of Table 2, as explained in Section 2.3.2-(2). The CV observed across this whole population showed very uneven results for the $\delta_{MA}$ (71.2%) and the ${MA}_{avg}$ (0.8%).

**Figure 12:**
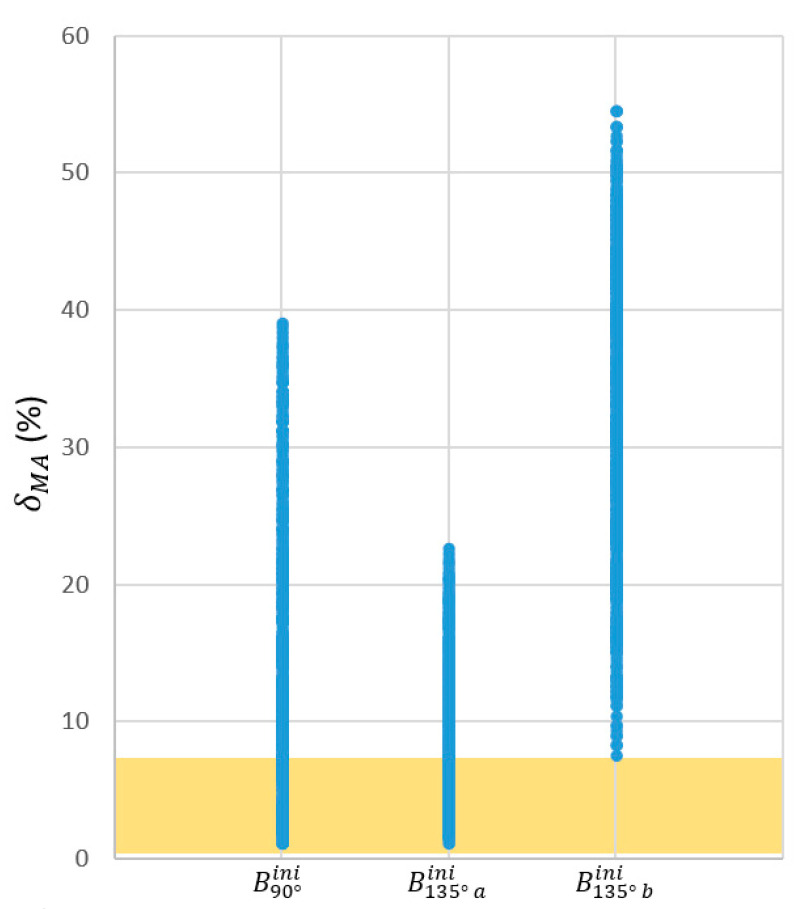
Dispersion of the different $\delta_{MA}$ of the ${3\cdot 3}^{6}=2187$ mechanisms resulting from an incremental, null, or decremental variation of the six parameters of Table 2 in those three selected cases which resulted from the optimization of the location of the joint $B^{ini}$ (resumed in Table 3). The colored region highlights the quartile of the population with the lowest $\delta_{MA}$, resulting in 547 proposals of RHEx.

Figure 12 summarizes the dispersion of the different $\delta_{MA}$. The region containing the first quartile of the population arranged in increasing order of $\delta_{MA}$ is highlighted, i.e., 547 versions of the mechanism with the lower $\delta_{MA}$. In the figure, these populations are grouped after their $B^{ini}$ for the sake of brevity. Basically, they include versions of the original mechanisms documented in Table 2 as $B_{90{}^{\circ}}^{ini}$ and $B_{135{}^{\circ}a}^{ini}$.

Among those pre-selected and ordered by their ${MA}_{avg}$, the $B_{90{}^{\circ}}^{ini}$ versions ranked the best 218 proposals. It is noteworthy that the very low CV shown by the ${MA}_{avg}$ implied an improvement of only $8.5\cdot 10^{-5}$ in that metric when choosing the mechanism with better ${MA}_{avg}$ instead the one with better $\delta_{MA}$. However, this change would imply a worsening of $\delta_{MA}$ of 6.7%. For that reason, the version of $B_{90{}^{\circ}}^{ini}$ which ranked better at the pre-selection based on the $\delta_{MA}$ was the one selected to achieve the RHEx (Figure 11d). Values for this mechanism are shown in Table 4.

### 3.3. Study of Correlations

#### 3.3.1. Study of Correlations in the Location of the Joint B

In Section 2.3.2-(1), a first location of $B^{ini}$ was proposed as the midpoint of the segment $S^{ini}O$ and then repositioned to five different locations, shown in Figure 7a. For each hypothesis, four mechanisms were generated. In total, 20 mechanisms were available at the various $B^{ini}$ locations, whose quality metrics (${MA}_{avg}$ and $\delta_{MA}$) could be contrasted with those of the 4 mechanisms at the first $B^{ini}$ location. This resulted in 80 possible combinations on which to perform a correlation study between the variation of the quality metrics and the relocation of $B^{ini}$.

Overall, no correlation was observed between $B^{ini}$ location and the variation in the value of $\delta_{MA}$ (−0.125 correlation coefficient). However, it is presumed that the location of $B^{ini}$ can be correlated with the variation in the value of ${MA}_{avg}$ (0.732 correlation coefficient).

The average observed variation of the ${MA}_{avg}$, for each possible direction for the relocation of $B^{ini}$, was as described in the Table 5, with the largest increases for the 135° and 90° directions. The results are consistent with the preselection made in Section 3.1.

#### 3.3.2. Study of Correlations in the Design Parameters

With the 2187 mechanisms explained in Section 3.2., a correlation study was carried out between each of the variables in Table 2 and the quality metrics used, ${MA}_{avg}$ and $\delta_{MA}$. Specifically, we studied the correlation between the variation in each of the varied parameters with respect to those in the original mechanism (resulting from the calculations in Section 2.3.2-(1)) and the variations in the metrics concerning the metric values in that same mechanism.

Positive values, in general, might indicate a direct relationship, i.e., when the parameter increases, the metric also tends to increase. This was the case with $L_{3p}$ concerning ${MA}_{avg}$ and $\delta_{MA}$ but the relationship was negligible. Also, $L_{3d}$ had a weak positive correlation with $\delta_{MA}$. However, $L_{3d}$ had a weak negative correlation with ${MA}_{avg}$. Regarding the angular parameters, $\varphi_{2p}$ had a very weak negative correlation with ${MA}_{avg}$ and with $\delta_{MA}$. On the other hand, $\varphi_{2d}$ had correlations very close to 0 with both metrics, thus dismissing any linear relationship between $\varphi_{2d}$ and these metrics.

In summary, all correlations summarized in Table 6 were weak, suggesting that there was no linear relationship between the parameters $L_{3p}$, $\varphi_{2p}$, $L_{3d}$, and $\varphi_{2d}$ with the quality metrics ${MA}_{avg}$ and $\delta_{MA}$.

### 3.4. Final Model

Taking into account that the ${MA}_{avg}$ values did show a correlation with the relocation of $B^{ini}$ in the 90° direction (see Figure 7a), a new simulation was performed in which the distance (radius) with respect to the first location of $B^{ini}$ was increased to 10 mm. We simulated not only the mechanism resulting from maintaining the parameters $L_{3p},\varphi_{2p},\varphi_{1p},L_{3d}$, $\varphi_{2d}$, and $\varphi_{1d}$ of the mechanism selected in Section 3.2 (values shown in Table 4) but also the 729 mechanisms resulting from variations of these variables. The mechanism directly obtained by displacing the $B^{ini}$ point had a ${MA}_{avg}$ of 0.0634 ($\delta_{MA}=$ 4.919). For the 729 variations, the ${MA}_{avg}$ had a mean value of 0.102 (0.063 min, 0.898 max, CV=123.11) and the $\delta_{MA}$ had a mean value of 109.94 (1.554 min, 741.190 max, CV=171.895). Note that the best ${MA}_{avg}$ had the highest $\delta_{MA}$ values associated with them. In the end, after a screening performed according to the procedure described in Section 3.2, the dimensions of the mechanism described in Table 7 were adopted for the design of the RHEx.

After the dimensional synthesis of the RHEx adapted to the patient’s anatomy, there is still room for the choice and location of the actuator. The basis for its installation has already been discussed in Section 2.3.3. With the assumption of distance OA=10 mm with which the mechanical advantage has been calculated, observing Equation (8), with τ=29.308° in the final design, it is found that the stroke length required in the linear actuator would be $L_{chord}=5.059$ mm. These calculations must be confronted with the reality of the actuators available on the market. For this purpose, the duration of the pulp pinch closing sequence was first estimated to be 1 s, applying a force of 5 N (see Section 1.1). Approximating the distance traveled by T to be about 40 mm, measured as the distance between the patient’s index fingertip and the end of the thumb, this implied a power requirement ($H_{T}$) of

(9) $$H_{T}=5\;N\cdot \frac{40\cdot 10^{-3}\;m}{1\;s}=0.2\;W.$$

The actuator selected should not provide less power than that of Equation (9): the model chosen for the development was the Actuonix^®^ PQ12 (maximum load of 50 N, 20 mm stroke length (i.e., available $L_{chord}$), 10 mm/s max speed [65]).

Furthermore, recalling Equation (3), it is observed that if the distance OA is multiplied by a factor k, the overall mechanical advantage of the mechanism varies by the same factor:

(10) $$∆s_{A}^{\prime }=k\cdot ∆s_{A}\to MA^{\prime }\approx \frac{k\cdot ∆s_{A}}{∆s_{T}}=k\cdot MA.$$

Recalling Equation (8), the radius OA taking benefit from the stroke length of the abovementioned actuator is OA=39.528 mm, and, following Equation (10) with k≈39.528/10, the averaged mechanical advantage for the final design of RHEx is ${MA}_{avg}^{\prime }=0.249$. All requirements were met:

(11) $$H_{A}=50\;N\cdot 0.010\;\frac{m}{s}=0.5\;W>0.2\;W,$$

(12) $$F_{T}{=N}_{AC}\cdot {MA}_{avg}^{\prime }=50\;N\cdot 0.249=12.45\;N>5\;N.$$

**Figure 13:**
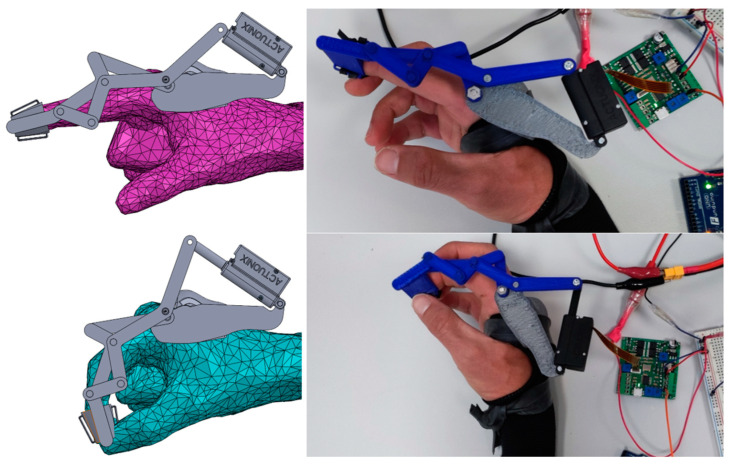
Final design and testing of the RHEx.

In Equation (12), the value of MA has been considered as that of ${MA}_{avg}$, given the low value of $\delta_{MA}$. Figure 13 shows the final RHEx design with the dimensions resulting from the optimization process and with the selected linear actuator. The RHEx was modeled in SolidWorks^®^ 2025 with the dimensions obtained after optimization in Matlab^®^R2018b, and later, it was printed in Acrylonitrile Butadiene Styrene (ABS) via Fused Deposition Modeling (FDM) on a 3D printer, namely, a CoLiDo^®^ mod. X3045 (CoLiDo Ibérica, Valencia, Spain) with Repetier-Host v2.3.2 (www.repetier.com accessed on 10 June 2024) software. The proposed assembly is grounded on the dorsum of the palm, and it avoids any interference with it during the movement.

## 4. Discussion

The focus on improving the quality of life and rehabilitation prospects for post-stroke patients has become increasingly significant in recent years. Hand exoskeletons, including assistive ones, should be as compact and lightweight as possible to enhance wearability. Underactuated designs, like the one examined in this study, are well-suited for this goal, as they aim for compact actuator sizes while delivering high output forces (relative to the actuator’s size) and ensuring efficient power transmission through the links. Despite progress, research on exoskeletons with enhanced mechanical advantages and ergonomics still requires revisions and refinements to meet these specific needs. The scanning process, along with the mechanism optimization presented here, are essential tools for developing custom-made, optimal mechanisms tailored to each patient.

The results of this research have focused on a 6-bar topology which is versatile for the design of exoskeletons both for the whole hand (in the case of a dorsal layout) and for index finger rehabilitation, as has been the case (with a more compact lateral layout).

The integration of design and optimization techniques raises an important question: how do engineers determine the most suitable method or algorithm for optimizing different design criteria. To address these challenges, alternative optimization approaches have been developed, including nature-inspired methods from evolutionary computation (EC). The strategy followed in this research provides design procedures applicable to similar exoskeletons. First, the search for the optimal location for the concatenation joint of the four-bar mechanisms (B-joint), and second, the search for correlations on the design variables (the location coordinates of the B-joint included). We have worked with quality metrics that are of interest for the performance of the task, such as ${MA}_{avg}$ and $\delta_{MA}$. It should be noted that the lack of correlations between the variation of the design parameters and the quality metrics evidenced the high non-linearity of the system. Only the coordinates of the B-joint showed some significance. All things considered, these metrics have allowed us to obtain a mechanism with a quasi-constant MA value across its travel, with an average value (${MA}_{avg}$) higher than the result of our previous studies [45,46] mentioned earlier (this former design had values of MA=0.064 at the final posture and MA=0.051 at the initial posture, calculated according to Equation (3), with $\delta_{MA}=22.6\%$).

By targeting genetic optimization schemes, we reduced the number of iterative or generational calculations required to reach an optimal solution. In this case, compared to a previous optimization for the same problem [45,46], we only evaluated 2211 different mechanisms, instead of 59,049. This signifies the elimination of 96.2% of the cases that were randomly generated in those studies.

## 5. Conclusions

The selection and design of the best topologies for the design of collaborative or rehabilitative exoskeletons is a growing field because their applications in modern life have been boosted in parallel to the explosion of control techniques and skin-interfacing flexible electronics (e-skin). Moreover, the integration of artificial intelligence in these systems could further enhance their functionality, enabling predictive diagnostics and personalized rehabilitation strategies based on real-time data analysis. This would make rehabilitation more efficient and accessible to patients both in clinical settings and at home. However, the optimization of the topologies to be used in the kinematic chain is crucial; therefore, the choice of good optimization functions must ensure that they evaluate the mechanism throughout its functional range.

The targeted genetic optimization scheme here exposed, by observing the ${MA}_{avg}$ and $\delta_{MA}$ and their relationship with specific parameters identified in the different stages of the mechanism synthesis, allows us to arrive at designs that improve the overall behavior of the RHEx. On the one hand, this has made it possible to reduce the computational cost which can be abused with current means but would not justify a good selection process. On the other hand, this optimization can be easily applied to any other exoskeleton after studying its functionality.

The following conclusions can be made on the resulting prototype of RHEx:

- The movement of the RHEx is custom-made and adequate for the patient’s requirements. Being an underactuated mechanism, it can be actuated by one small linear actuator. The power supply of the prototype is a low-voltage battery (up to 12 Volts) which poses no risk to the user.
- It is verified that the RHEx has a low weight and that the fastening system to the hand is not uncomfortable.
- Regarding the safety of the prototype and future work, the following can be taken into account:
- To leave the fingertip free, designs that adhere the final segment of the RHEx to the nail and sides of the patient’s distal phalanx may be considered.
- The same procedure can be adapted and replicated for an RHEx design allowing flexion of the thumb, which now remains fixed in opposition.

The RHEx design protocol here presented, from conception to optimization, is adaptable to any hand size, ensuring that institutions can effectively serve a wide range of patients during rehabilitation. By utilizing 3D printing technology based on a precise scan of the patient’s hand, it becomes possible to create exoskeletons that are perfectly tailored to individual hand dimensions. This level of customization enhances both fit and comfort, which is challenging to achieve with conventional, one-size-fits-all devices [66].

Additionally, 3D printing allows healthcare facilities to produce these devices in-house at a very low cost, using materials like PLA or ABS, which are both affordable and recyclable [67,68]. This approach not only ensures that the developed technology is accessible to patients with different hand sizes but also reduces costs and delivery times by enabling on-site production.

## Figures and Tables

**Table 1:** Hand exoskeletons optimized with evolutionary (EC) or non-evolutionary (nEC) computation techniques in terms of application (assistance (AHEx), rehabilitation (RHEx), or haptic (HHEx)), optimization metrics, and optimization method (2014–2023).

Authors	Application	Metrics ^1^	Method
Li et al. [22]	AHEx	FT	Elitist Non-Dominated Sorting Genetic Algorithm (EC) [28,29]
Du et al. [23]	RHEx	FT	Single-Objective Genetic Algorithm (EC) [30]
Iqbal et al. [24]	RHEx	KM, CA, GI, FT	Weight-Based Genetic Algorithm (EC) [21]
Vanteddu et al. [25]	AHEx	AC	Weight-Based Genetic Algorithm (EC) [21]
Li et al. [26]	RHEx	M	Elitist Non-Dominated Sorting Genetic Algorithm (EC) [28,29]
Sarac et al. [27]	AHEx	FT	Single-Objective Genetic Algorithm (EC) [30]
Amirpour et al. [31]	HHEx	W, AC	Levenberg–Marquardt Algorithm (nEC) [32,33]
Bianchi et al. [34]	RHEx	FT, S	Levenberg–Marquardt Algorithm (nEC) [32,33]
Liang et al. [35]	AHEx, RHEx	W	Geometric differentiation (nEC) [36]
Xu et al. [37]	RHEx	W	Interior point algorithm (nEC) [38]
Qin et al. [39]	RHEx	AC	Goal attainment method (nEC) [40]
Secciani et al. [41]	AHEx	AC	Interior point algorithm (nEC) [38]

^1^ FT: force transmission; W: workspace; S: size; AC: adjustability/calibration; KM: kinematic mapping; CA: collision avoidance; GI: global isotropy index; MI: manipulability index.

**Table 2:** Parameters on which to perform variations across the different tests.

	$L_{3p}$	$\varphi_{2p}$	$\varphi_{1p}$	$L_{3d}$	$\varphi_{2d}$	$\varphi_{1d}$
Min.	10 mm	90°	230°	4 mm	90°	230°
Max.	25 mm	170°	340°	8 mm	170°	340°

**Table 3:** Dimensions and values of $\delta_{MA}(\%)$ and ${MA}_{avg}$ for the six preselected cases from the optimization of the location of the joint $B^{ini}$. Lengths (*L*) in mm, angles (φ) in *deg*, $\delta_{MA}$ in %.

	$L_{1p}$	$L_{2p}$	$L_{3p}$	$L_{4p}$	$\varphi_{1p}$	$\varphi_{2p}$	$L_{1d}$	$L_{2d}$	$L_{3d}$	$L_{4d}$	$\varphi_{1d}$	$\varphi_{2d}$	$\delta_{MA}$	${MA}_{avg}$
$B_{135{}^{\circ}a}^{ini}$	8.995	38.290	17.755	42.741	250.575	98.641	21.534	31.256	4.018	45.738	334.882	134.750	1.881	0.056
$B_{90{}^{\circ}}^{ini}$	8.406	34.950	15.141	42.827	292.691	109.698	15.831	34.950	4.537	21.696	247.841	156.550	4.292	0.057
$B_{180{}^{\circ}}^{ini}$	13.169	39.591	11.140	35.899	266.028	122.145	6.838	29.591	4.736	30.169	285.022	99.866	10.005	0.048
$B_{45{}^{\circ}a}^{ini}$	8.620	31.256	15.084	37.617	273.232	104.319	14.883	38.290	5.563	30.328	248.439	98.123	18.783	0.052
$B_{45{}^{\circ}b}^{ini}$	8.076	31.256	14.555	33.955	236.341	94.383	39.015	38.290	6.881	70.426	334.430	105.638	25.161	0.052
$B_{135{}^{\circ}b}^{ini}$	9.819	38.290	14.746	51.646	313.820	90.096	18.820	31.256	4.493	40.292	321.311	141.643	30.965	0.057

**Table 4:** Best mechanism after the optimization of the design parameters. The values shown for the version of $B_{90{}^{\circ}}^{ini}$ were taken for the design of the RHEx. Lengths (***L***) in mm, angles (φ) in *deg*, $\delta_{MA}$ in %.

	$L_{1p}$	$L_{2p}$	$L_{3p}$	$L_{4p}$	$\varphi_{1p}$	$\varphi_{2p}$	$L_{1d}$	$L_{2d}$	$L_{3d}$	$L_{4d}$	$\varphi_{1d}$	$\varphi_{2d}$	$\delta_{MA}$	${MA}_{avg}$
$B_{90{}^{\circ}}^{ini}$	8.796	34.950	17.141	45.379	292.691	104.698	15.247	34.950	4.537	23.158	252.841	156.550	0.884	0.057

**Table 5:** Average variation in the ${MA}_{avg}$ for each of the directions of the displacement of $B^{ini}$ depicted in Figure 7.

Dir. Displacement $B^{ini}$	$∆{MA}_{avg}$
0°	−0.0046
45°	0.0079
90°	0.0131
135°	0.0117
180°	0.0038

**Table 6:** Correlation coefficients between the design parameters varied and the quality metrics.

	$L_{3p}$	$\varphi_{2p}$	$L_{3d}$	$\varphi_{2d}$
${MA}_{avg}$	0.0634	−0.0155	−0.1893	0.0024
$\delta_{MA}$	0.0808	−0.0137	0.1650	−0.0148

**Table 7:** Dimensions taken for the final design of the RHEx. Lengths (*L*) in mm, angles (φ) in *deg*, $\delta_{MA}$ in %.

	$L_{1p}$	$L_{2p}$	$L_{3p}$	$L_{4p}$	$\varphi_{1p}$	$\varphi_{2p}$	$L_{1d}$	$L_{2d}$	$L_{3d}$	$L_{4d}$	$\varphi_{1d}$	$\varphi_{2d}$	$\delta_{MA}$	${MA}_{avg}$
$B_{90{}^{\circ}}^{ini}$	3.650	36.007	15.141	46.176	286.665	99.698	25.122	36.007	4.537	19.513	252.522	156.550	1.554	0.063

## Data Availability

The biometrics and the programming code in Matlab^®^ that supported the findings of this research are located in controlled access data storage at the Biomechanics and Ergonomics repositories of the Universitat Jaume I. The data used in this study are available on request from the corresponding author due to sensitivity reasons.
